# Comprehensive analysis of mitophagy-related genes in diagnosis and heterogeneous endothelial cells in chronic rhinosinusitis: based on bulk and single-cell RNA sequencing data

**DOI:** 10.3389/fgene.2023.1228028

**Published:** 2023-09-08

**Authors:** Shican Zhou, Kai Fan, Ju Lai, Shiwang Tan, Zimu Zhang, Jingwen Li, Xiayue Xu, Chunyan Yao, BoJin Long, Chuanliang Zhao, Shaoqing Yu

**Affiliations:** ^1^ Department of Otorhinolaryngology-Head and Neck Surgery, Tongji Hospital, School of Medicine, Tongji University, Shanghai, China; ^2^ Department of Allergy, Tongji Hospital, School of Medicine, Tongji University, Shanghai, China

**Keywords:** chronic rhinosinusitis, mitophagy, endothelial cells, single-cell RNA analysis, bulk RNA analysis

## Abstract

**Background:** Chronic rhinosinusitis (CRS) is a complex inflammatory disorder affecting the nasal and paranasal sinuses. Mitophagy, the process of selective mitochondrial degradation via autophagy, is crucial for maintaining cellular balance. However, the role of mitophagy in CRS is not well-studied. This research aims to examine the role of mitophagy-related genes (MRGs) in CRS, with a particular focus on the heterogeneity of endothelial cells (ECs).

**Methods:** We employed both bulk and single-cell RNA sequencing data to investigate the role of MRGs in CRS. We compiled a combined database of 92 CRS samples and 35 healthy control samples from the Gene Expression Omnibus (GEO) database and we explored the differential expression of MRGs between them. A logistic regression model was built based on seven key genes identified through Random Forests and Support Vector Machines - Recursive Feature Elimination (SVM-RFE). Consensus cluster analysis was used to categorize CRS patients based on MRG expression patterns and weighted gene co-expression network analysis (WGCNA) was performed to find modules of highly correlated genes of the different clusters. Single-cell RNA sequencing data was utilized to analyze MRGs and EC heterogeneity in CRS.

**Results:** Seven hub genes—SQSTM1, SRC, UBA52, MFN2, UBC, RPS27A, and ATG12—showed differential expression between two groups. A diagnostic model based on hub genes showed excellent prognostic accuracy. A strong positive correlation was found between the seven hub MRGs and resting dendritic cells, while a significant negative correlation was observed with mast cells and CD8^+^ T cells. CRS could be divided into two subclusters based on MRG expression patterns. WGCNA analysis identified modules of highly correlated genes of these two different subclusters. At the single-cell level, two types of venous ECs with different MRG scores were identified, suggesting their varying roles in CRS pathogenesis, especially in the non-eosinophilic CRS subtype.

**Conclusion:** Our comprehensive study of CRS reveals the significant role of MRGs and underscores the heterogeneity of ECs. We highlighted the importance of Migration Inhibitory Factor (MIF) and TGFb pathways in mediating the effects of mitophagy, particularly the MIF. Overall, our findings enhance the understanding of mitophagy in CRS, providing a foundation for future research and potential therapeutic developments.

## 1 Introduction

Chronic rhinosinusitis (CRS) is a prevalent and debilitating condition that substantially impacts the quality of life of affected individuals (Lal et al., 2023). It is estimated to affect approximately 5%–15% of the general population worldwide, leading to significant morbidity and economic burden (Rudmik, 2015; Liu et al., 2020; Naclerio et al., 2023). The underlying pathophysiology of CRS is multifactorial and complex, involving a combination of host, environmental, and microbial factors (Qureshi et al., 2023). CRS is typically characterized by inflammation of the nasal and paranasal sinus mucosa lasting for more than 12 weeks (Kim et al., 2022). Its symptoms encompass a range of manifestations, including nasal obstruction, rhinorrhea, facial pain or pressure, and a decrease in sense of smell (Al-Ahmad et al., 2022). The etiology of CRS is not fully understood, but the disease is believed to be a result of a dysregulated immune response to environmental and microbial stimuli in a genetically susceptible host (Lee and Lane, 2011). The disease is often categorized according to the presence or absence of nasal polyps (CRSwNP and CRSsNP, respectively), each presenting distinct pathological and clinical features (Xuan et al., 2022). Further to this, based on the degree of eosinophilic cells infiltration, CRSwNP is stratified into eosinophilic chronic rhinosinusitis with nasal polyps (ECRSwNP) and non-eosinophilic chronic rhinosinusitis with nasal polyps (nECRSwNP) (Lou et al., 2018). ECRSwNP is often associated with a Type 2 inflammatory response characterized by eosinophilia, elevated levels of IL-5, IL-13, and IgE, whereas nECRSwNP typically shows a mixed or Type 1 inflammatory pattern (Li et al., 2021; Shen et al., 2022).

Mitophagy, a specialized subset of autophagy, selectively degrades and eliminates damaged or dysfunctional mitochondria under various stress conditions such as hypoxia, depolarization, and infectious challenges (Mishra and Thakur, 2023). Mitochondrial integrity is crucial for cellular energy supply and homeostasis, and its disruption can lead to impaired bioenergetics, redox control, and ultimately, cell death (Harrington et al., 2023). The mitophagic process involves specific receptors that identify defective mitochondria, initiating their encapsulation by an isolation membrane or phagophore for subsequent lysosomal degradation (De et al., 2021). This process is governed by diverse signaling pathways, including PINK1/Parkin, BNIP3/Nix, and FUNDC1, with their involvement varying according to cell type (Iorio et al., 2021). Impaired mitophagy is associated with a wide range of diseases, including cancers, heart failure, and neurological disorders (Liu H. et al., 2022). In the field of pulmonary diseases, the implications of mitophagy have been explored in conditions such as Chronic Obstructive Pulmonary Disease (COPD), Asthma, and potentially Acute Respiratory Distress Syndrome (ARDS) (Araya et al., 2019; Li et al., 2019; Zhang et al., 2019). Nevertheless, the involvement of mitophagy in Chronic Rhinosinusitis (CRS) remains less extensively investigated. This paper, therefore, aims to utilize the bulk and single-cell RNA sequencing data to probe the role of mitophagy-related genes (MRGs) in the pathogenesis of CRS. Particularly, we aim to explore how the differential expression of MRGs in various cell types might contribute to disease severity and heterogeneity, and whether these could serve as potential therapeutic targets for CRS. This approach may provide more precise diagnostic and therapeutic strategies, thereby improving the management and prognosis of patients with CRS.

## 2 Materials and methods

### 2.1 Identification of MRGs expression in nasal mucosa between CRS and healthy control group

**FIGURE 1:**
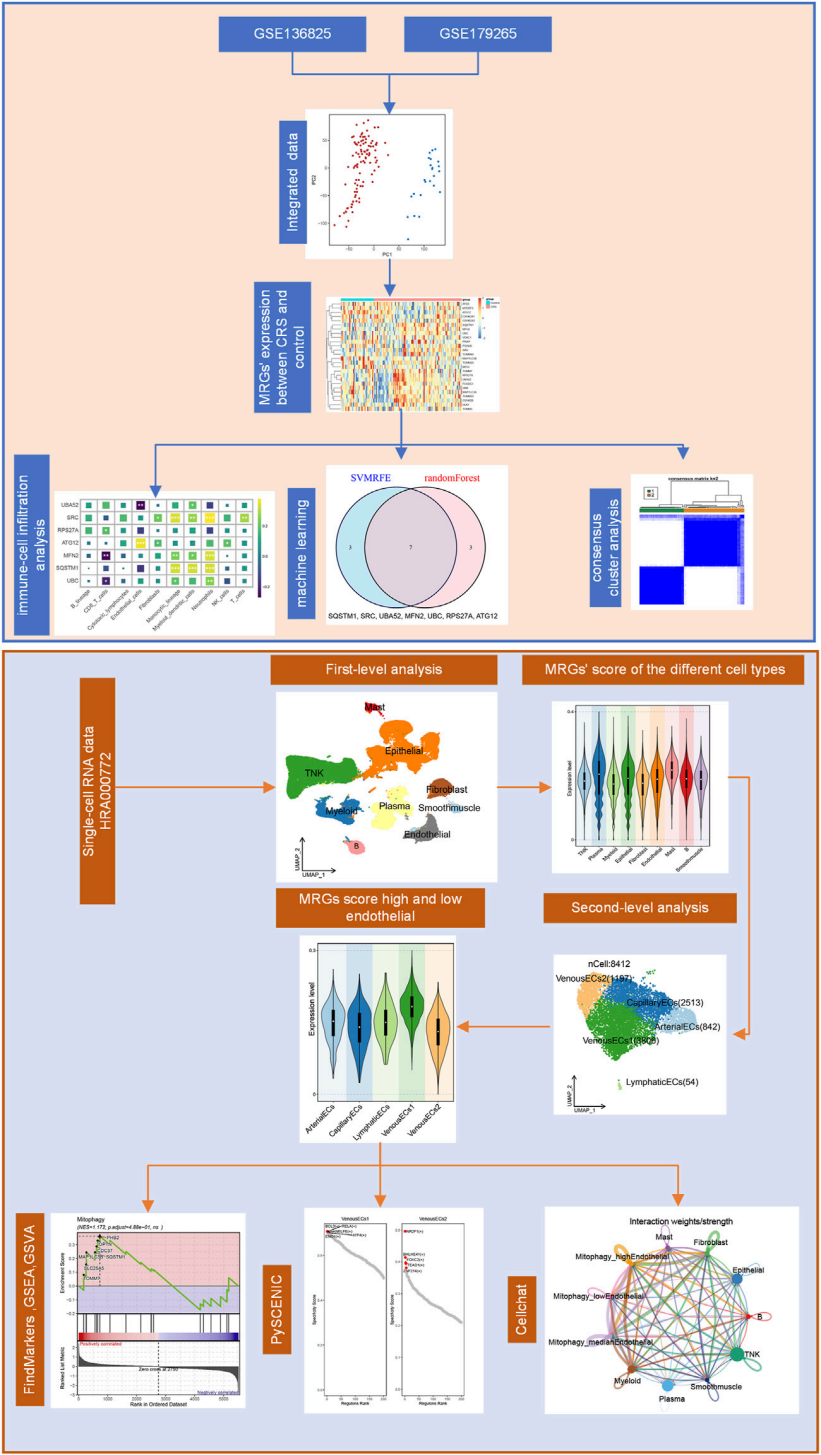
Flowchart for Comprehensive analysis of MRGs in diagnosis and heterogeneous ECs in CRS.

The detailed work flow is indicated in Figure 1. We obtained two gene expression matrix of human nasal mucosa (GSE136825, GSE179265) from the Gene Expression Omnibus (GEO) database (https://www.ncbi.nlm.nih.gov/geo/). A merged database of 92 CRS samples and 35 healthy control (Control) samples were obtained by de-batch-based operation with sva packages in R software. The 29 MRGs were obtained from Reactome (https://reactome.org/), and the difference in these gene’s overall expression between CRS and Control was then compared using “limma” package.

### 2.2 Identification hub MRGs in CRS

To identify the hub genes in CRS, random forest classifiers and SVM recursive feature elimination (SVM-RFE) analyses were implemented using the “randomForest” and “SVM-RFE” R packages. Each MRGs were ranked by importance based on random forest analysis and the SVM-RFE algorithm, and the top 10 genes were reserved. Then, the MRGs were identified by taking the intersection of the top 10 MRGs ranked by random forest and SVM-RFE analysis separately.

### 2.3 Construction of receiver operating characteristic curve and nomogram

The hub genes were subjected to multivariate logistic regression analysis, and the area under the receiver operating characteristic (ROC) curve (AUC) was calculated to evaluate their diagnostic value in CRS by using “pROC” package. A nomogram was developed to predict the possibility of CRS, and the calibration curve and decision curve analyses were drawn to present the stability of the model. The model was retested for internal validation using bootstrap, with 1000 bootstrap replicates. We evaluated the discrimination using the AUC of the ROC curve. An AUC >0.80 was considered to be an acceptable value. Model calibration was assessed using the Hosmer–Lemeshow goodness-of-fit test. Decision curve analysis (DCA) was used to assess the clinical usefulness of the logistic regression model.

### 2.4 The relationship between the hub MRGs and immune cells infiltration

We utilized the IODR package for a comprehensive immunoinfiltration analysis (Zeng et al., 2021). This tool allowed us to explore the relationships between our chosen hub MRGs and various immune cell types, which included dendritic cells (DC), mast cells, and CD8^+^ T cells, among others. Furthermore, we implemented the MCPcounter technique for additional immunoinfiltration analysis (Becht et al., 2016). This tool enabled us to quantify the relative abundance of immune infiltrates from the gene expression profiles, providing insight into the association between the hub MRGs and immune cells like neutrophils, monocytes, and myeloid cells. Besides, we conducted a correlation analysis to assess the relationship between the seven diagnostic MRGs and the inflammatory factors. For this purpose, we used the Pearson correlation coefficient to establish the degree and direction of the association.

### 2.5 Consensus clustering analysis and co-expression analysis

Consensus cluster analysis was operated using “ConsensusClusterPlus”, and the maximum number of cluster genes in CRS samples was set as 10. The top 5000 most variable genes were measured and clustered CRS samples by a median absolute deviation. Unsupervised consensus clustering was operated to cluster CRS samples and identify the best number of optimal clusters (Monti et al., 2003). The weighted gene co-expression network analysis (WGCNA) package in R (Langfelder and Horvath, 2008). Was used to identify sets of genes with similar mRNA expression profiles across CRS samples using the default parameters. The scale-free topology (SFT) criterion was used to choose the soft threshold parameter of the power adjacency function, and the optimal threshold parameter value was accepted based on the SFT criterion recommendation (model-fit saturation > 0.85).

### 2.6 GO, KRGG and GSVA function analysis

We utilized the Molecular Signatures Database (MSigDB) to acquire gene sets associated with *Homo sapiens* in the “Hallmark” category using the “msigdbr” R package. Using “clusterProfile” packages (Yu et al., 2012) in R, GO, and Kyoto Encyclopedia of Genes and Genomes (KEGG) pathway analyses were also conducted. We also use Metascape (Zhou et al., 2019) for enrichment analysis. For the computation of gene set variation analysis (GSVA), we utilized the “GSVA” package in R and selected the single-sample gene set enrichment analysis (ssGSEA) method with a Gaussian kernel cumulative distribution function (Hänzelmann et al., 2013).

### 2.7 scRNA-seq analysis

#### 2.7.1 Basic analysis workflow of scRNA-seq data

We retained cells that expressed a minimum of 300 genes and had mitochondrial gene counts constituting less than 20% of total gene counts for quality control of scRNA-seq data. Each cell was assigned a cell cycle stage using the CellCycleScoring function in the Seurat package (version 4.3.0) (Stuart et al., 2019). Data normalization was performed using the scTransform function, which incorporated the S. Score and G2M.Score. Dimension reduction and clustering analysis were executed using the Seurat package. To mitigate the batch effect, which may impede the accuracy of single cell analysis, we implemented batch effect correction analysis using the harmony package (version 0.1.1) based on the top 3000 variable genes with the default harmony parameters. The selection of the principal components (PCs) was informed by both elbow and Jackstraw plots. For clustering, we employed the FindNeighbors and FindClusters functions, which implement a shared nearest neighbor (SNN) modularity optimization-based clustering algorithm on the identified number of principal components with a resolution of 0.8. The clusters were visualized by UMAP using Seurat’s RunUMAP function. The clusters were assigned identifiers based on the unique gene expression profile of each cluster. We calculated MRGs score using UCell V.2.2.0 (Andreatta and Carmona, 2021). Differential gene expression analysis Differential gene expression analysis was performed using FindMarkers function with default parameters. We followed the same procedure for the secondary clustering of ECs, with a resolution of 0.2.

#### 2.7.2 Construction and analysis of the transcription factor-gene network

We employed pySCENIC (Aibar et al., 2017; Van de Sande et al., 2020) for the identification of transcription factor regulons. A count matrix was constructed using 10,000 variable genes selected. Genes that were expressed in less than 1% of cells were excluded in accordance with the recommendations of the pySCENIC protocol. The gene co-expression network was deduced using the gradient boosting machine, that is, implemented in Arboreto. Enriched motifs for each gene co-expression module were predicted using the pre-computed databases from cisTargetDB and the ctx function in pySCENIC. Lastly, the activity scores of the inferred regulons were quantified at the single-cell level using AUCell.

#### 2.7.3 Cell-cell communication analysis

Cell–cell communication analysis was performed using the R CellChat (v 1.6.1) package (Jin et al., 2021). Initially, the normalized expression matrix was imported into the CellChat object using the “createCellChat” function. Subsequently, the data underwent preprocessing utilizing default parameters in the “identifyOverExpressedGenes”, “identifyOverExpressedInteractions”, and “projectData” functions. Potential ligand–receptor interactions were then calculated using “computeCommunProb”, “filterCommunication” (min.cells = 10), and “computeCommunProbPathway” functions. Lastly, the cell communication network was consolidated utilizing the “aggregateNet” function.

### 2.8 Statistical analysis

All raw data processing was conducted in R software (version 4.2.1). Student’s t-test or Wilcoxon’s rank sum test was used to detecting the significant difference between two independent groups. Kruskal-Wallis-test was used to explore differences among more than two independent groups. All statistical *p* values were two-sided, *p* < 0.05 was considered statistically significant.

## 3 Results

### 3.1 Constructed upon the hub MRGs, the CRS diagnostic model was established

**FIGURE 2:**
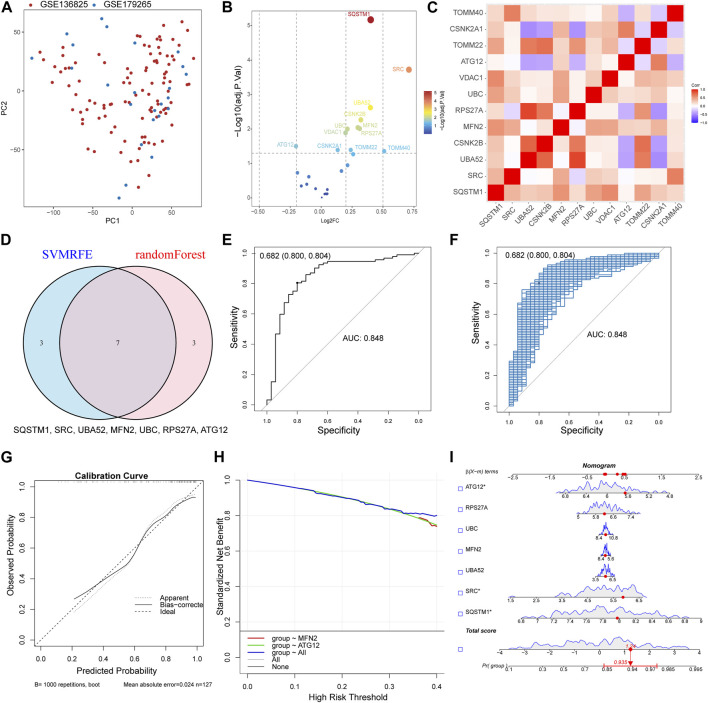
Constructed upon the hub MRGs, the CRS diagnostic model was established. **(A)** PCA plot of two obtained datasets after batch effects are removed. **(B)** Volcano plot of differentially expressed MRGs between CRS and Control. **(C)** Heat map of the differentially expressed MRGs correlation analysis. **(D)** Seven hub genes were identified by random forest and SVM-RFE analysis. **(E)** Receiver operating characteristic (ROC) curve of predicted risk scores in CRS diagnosis. **(F)** The AUC score for the full dataset was calculated and then 1000 bootstrap samples of the AUC score were used to obtain a confidence interval for each AUC score. **(G)** Calibration curve for the diagnostic model. **(H)** Comparison of decision curve analysis (DCA) of different genes. **(I)** Nomogram of seven hub MRGs in the diagnosis of a CRS sample.

The datasets GSE136825 and GSE179265 were processed through the sva R package for the refinement of batch effects and comprehensive data integration, leading to the procurement of 35 control subjects and 92 CRS cases (Supplementary Figure S1A). The PCA graph conclusively demonstrates the effective eradication of batch effects (Figure 2A). For the exploration of inter-group Differentially Expressed Genes (DEGs), the Linear Models for Microarray and RNA-Seq Data (limma) application was effectively employed. The detailed differential gene expression profiles are presented in Supplementary Table S1. The delineation of MRGs’ expression patterns spanning across diverse groups was achieved via a volcano plot, identifying genes such as SQSTM1, SRC, and UBA52 exhibiting a pronounced surge in CRS expression, whereas ATG12 demonstrated a conspicuously diminished expression (Figure 2B). Additional investigation into the correlation among MRGs unveiled a marked positive correlation between UBA52 and RPS27A, starkly juxtaposed by a significant inverse correlation between UBA52 and CSNK2A1 (Figure 2C).

By synergizing the capabilities of Random Forests and Support Vector Machines—Recursive Feature Elimination (SVM-RFE), a meticulous selection process for MRGs was enacted, with the confluence of selected genes unveiling the identification of seven hub genes (SQSTM1, SRC, UBA52, MFN2, UBC, RPS27A, and ATG12) (Figure 2D). Then, a logistic regression model was used to construct the prediction model base on the seven hub genes (Supplementary Figure S1B). The resultant model demonstrated commendable prognostic acuity (AUC = 0.848), as visually represented in Figure 2E. The subsequent stage involved corroborating the Receiver Operating Characteristic (ROC)’s reliability through a bootstrap method, incorporating random samples (*n* = 1000 bootstraps) with replacement (Figure 2F). The calibration curve compellingly showcased the model’s stability (Figure 2G). Moreover, the aggregated distribution scope of the AUC, specificity, and sensitivity collectively vouch for the model’s robust diagnostic competency (Supplementary Figures S1C, D, E). The nomogram model transcended the diagnostic value of the individually designated gene (Figure 2H). The accurate diagnosis of patients was achieved through the application of this model (Figure 2I). The Chronic Rhinosinusitis (CRS) diagnostic model was established Constructed upon MRGs, suggesting that MRGs play an important role in the occurrence of CRS.

### 3.2 The expression of hub genes correlated with the immune cell and the inflammatory factors

**FIGURE 3:**
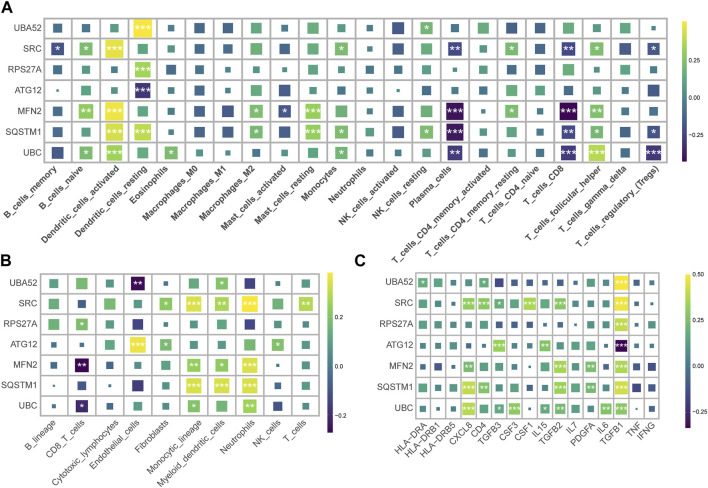
Correlation of hub MRGs with immune cell infiltration and inflammatory factors. **(A)** Graphical representation of the interrelation between seven hub MRGs and various immune cell types, as analyzed by the IODR package’s deconvo_cibersort function. **(B)** Using the IODR package’s deconvo_mcpcounter function to analyze the interrelation between seven hub MRGs and various immune cell types. **(C)** The correlation of the seven diagnostic MRGs with the inflammatory factor.

We harnessed the capabilities of the IODR package to perform an immune-cell infiltration analysis, which predominantly involved an exploration of the interrelations between seven hub MRGs and various immune cell types. The findings highlighted a robust positive interconnection between the seven hub MRGs and resting dendritic cells (DC), concurrently exhibiting a significant inverse correlation with mast cells and CD8^+^ T cells (Figure 3A). Simultaneously, we implemented the MCPcounter technique for immunoinfiltration analysis, where the seven hub MRGs displayed a substantial positive association with neutrophils, monocytes, and myeloid cells, contrasted by a distinct negative linkage with CD8^+^ T cells (Figure 3B).

An evaluation of the interconnectedness between the seven diagnostic MRGs and inflammatory factors suggested that the upregulated MRGs in CRS bore a positive correlation with TGFB1, while the downregulated MRGs in CRS demonstrated a negative correlation with TGFB1 (Figure 3C). This insinuates that TGFB1 may ostensibly assume a pivotal function within the realm of mitophagy.

### 3.3 Based on MRGs, CRS can be divided into two subclusters

**FIGURE 4:**
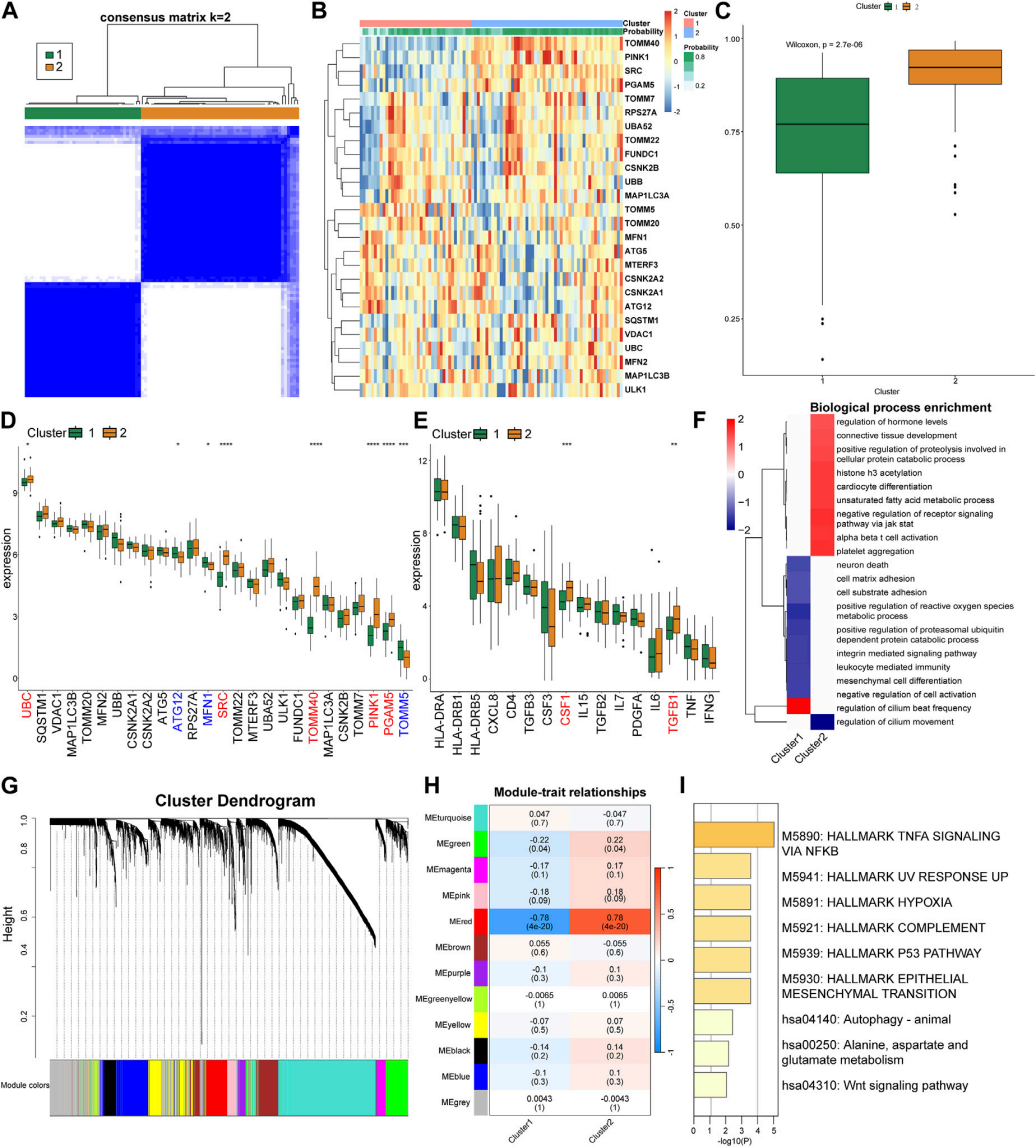
Cluster analysis and gene expression patterns of CRS patients based on MRGs. **(A)** Consensus matrix plots depicting consensus values on a white-to-blue color scale ordered by consensus clustering when two clusters were selected. **(B)** Heat map of the differentially expressed MRGs between the two cluster. **(C)** Differences in the predicted possibility of CRS among two clusters. **(D)** Differential expression of the MRGs in each cluster. **(E)** Differential expression of the inflammatory factors in each cluster. **(F)** GSEA pathway differential analysis showing activated and inhibited pathways in the two clusters. **(G)** Clustering dendrogram of genes based on topological overlapping. **(H)** Heatmap of the correlation between module eigengenes and the two clusters. **(I)** Enrichment analysis using Metascape for genes in the red module.

Based on the expression patterns of the MRGs, we grouped the CRS patients into two distinct clusters employing consensus cluster analysis (Figures 4A, B). Patients in Cluster 2 were more likely to be diagnosed with CRS (Figure 4C). Cluster 2 exhibited a conspicuous upregulation of UBC, SRC, TOMM40, PINK1, PGAM5, whilst a significant downregulation of MFN1, TOMM5, ATG12 was discerned (Figure 4D). Differences in the expression of inflammatory factors revealed that Cluster 2 exhibited elevated expression of CSF1 and TGFB1 (Figure 4E). Subsequently, we used GSEA for enrichment analysis, conducting a pathway differential analysis for the two clusters. The results indicated activation of pathways such as “negative regulation of receptor signaling pathway via JAK STAT” and “alpha beta T-cell activation” within the clusters, while the “regulation of cilium movement” pathway was inhibited (Figure 4F).

Next, we utilized WGCNA to explore the correlated genes of the two clusters. Quality control was performed on 92 CRS samples with none being removed (Supplementary Figure S2A). Co-expression modules were formulated utilizing dynamic tree-cut analysis, with the soft threshold set to 9 as an optimal SFT (scale-free topology) to construct a scale-free network (Supplementary Figure S2B). All statistically significant co-expression modules were identified based on optimal dynamic tree cut and hierarchical clustering (Figure 4G). In particular, the red module demonstrated a significant positive correlation with Cluster 2 and a significant negative correlation with Cluster 1 (*p* < 0.05) (Figure 4H). We subsequently used Metascape to perform enrichment analysis on the genes in the red module, which primarily identified pathways such as “TNFA signaling via NFKB”, “Hallmark Hypoxia”, and “P53 Pathway” (Figure 4I).

### 3.4 Single-cell analysis reveals that there are two types of endothelial MRGs score high and low in the nasal mucosa

**FIGURE 5:**
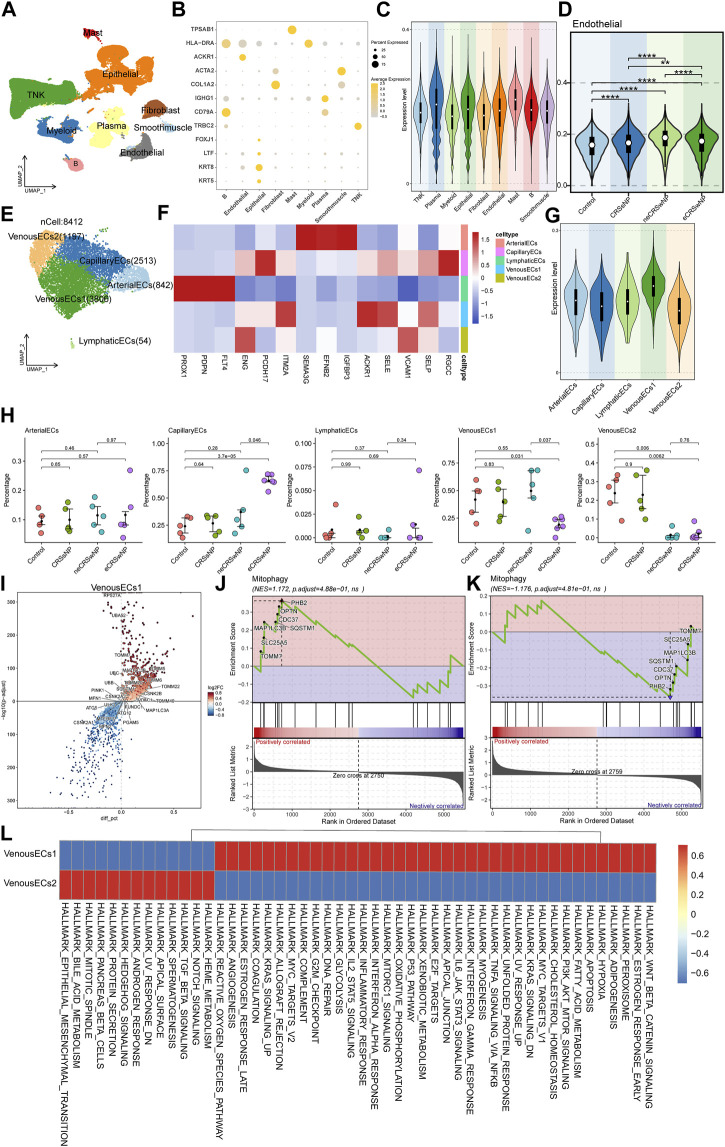
Single-cell analysis of MRGs in different cell types and detailed analysis of ECs. **(A)** UMAP plot illustrating nine identified first level cell types in the nasal mucosa. **(B)** Heat map of marker genes defining each cell type. **(C)** UCell scoring of MRGs for the different cell types. **(D)** MRGs score comparison of ECs among control and disease groups. **(E)** UMAP plot depicting five annotated EC types. **(F)** Heat map of marker genes defining each EC types. **(G)** UCell scoring of MRGs for each EC types. **(H)** Intergroup variation in cell proportions for each EC types. **(I)** Differential gene expression analysis between the two types of venous EC. **(J,K)** GSEA enrichment analysis showing activation of the mitophagy pathway in Venous EC1 and Venous EC2. **(L)** Functional enrichment analysis using GSVA.

To conduct an analysis related to MRGs at the single-cell level, we retrieved the original data HRA000772 from the Genome Sequence Archive, then using Cell Ranger to acquire the expression matrix. The permissions pertinent to data usage are comprehensively delineated in the attached document. This public study incorporated 21 samples, including Control, CRSsNP, neCRSwNP, and eCRSwNP, distributed as 5, 5, 5, and 6 samples, respectively. Upon initial quality control, we isolated a total of about 140,000 cells. Subsequently, we proceeded to perform batch correction, dimensionality reduction, clustering, and cell naming. By employing the “findallmark” function, we identified genes specific to each cell type (Supplementary Table S2), ultimately identifying nine cell types (Figure 5A). The marker genes, which served as the basis for naming each cell type, are depicted in Figure 5B. We then employed UCell to score the MRGs of the different cell types. The results illustrated that mast cells possessed the highest score, followed by plasma cells, with ECs also exhibiting a significant score (Figure 5C). Concurrently, the scoring results of ECs among different groups indicated that the control group had a lower score, while disease conditions elevated the score, particularly in the case of neCRSwNP (Figure 5D). The Ucell scores among groups for other cell types are shown in the (Supplementary Figure S3).

In our quest to further investigate the cause of variation in MRGs score of ECs among different groups, we extracted ECs for a secondary analysis. We annotated five cell types: arterial EC, capillary EC, lymphatic EC, and two types of venous EC (Figure 5E). The marker genes, which served as the basis for naming each cell type, are depicted in Figure 5F. Upon further implementation of UCell for MRGs scoring, we observed that the two types of venous EC displayed diametrically opposite scores (Figure 5G). The results of the intergroup variation in cell proportion demonstrated that venous EC1 with a high score had the highest proportion in neCRSwNP, while venous EC2 with a low score had the lowest proportion in neCRSwNP (Figure 5H). This alteration coincided with the scoring situation among different groups of ECs (Figure 5D), suggesting that the proportional difference between these two types of ECs might be the primary contributor to the variation in MRGs scores among endothelial cell groups.

We then proceeded to characterize these two types of venous EC. Using the “findmarker” function, we compared the differential genes between these two cell types, with the results detailed in Supplementary Table S3. The majority of mitochondria-related genes exhibited high expression in venous EC1 (Figure 5I). GSEA enrichment analysis also indicated the activation of the mitophagy pathway in venous EC1 (Figure 5J), and its suppression in venous EC2 (Figure 5K). The GSVA analysis revealed the variation in hallmark gene set enrichment across these two cell types. Notably, venous EC1 were enriched in pathways such as “Epithelial-Mesenchymal Transition”, “Notch Signaling”, and “Protein Secretion”, while venous EC2 endothelial cells displayed enrichment in the “Wnt Beta-Catenin Signaling”, “PI3K Akt mTOR Signaling”, and “IL6 Jak STAT3 Signaling” pathways (Figure 5L).

### 3.5 SCENIC analysis identified distinct transcriptional factor profiles in two type venous EC with high and low MRGs scores

**FIGURE 6:**
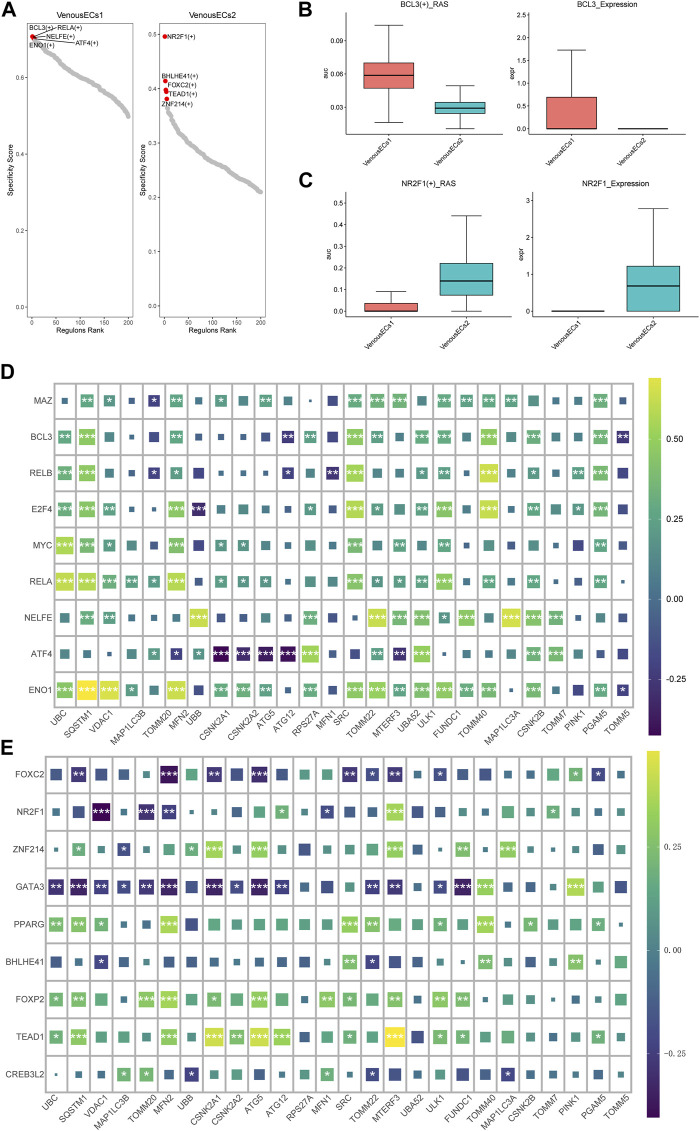
Transcription factor profiles and correlation analysis with MGRs in two types of venous EC. **(A)** Transcription factor profiles in venous EC1 and venous EC2. **(B)**The score of BCL3(+) _RAS and the expression of BCL3 in venous EC1 and venous EC2. **(C)**The score of NR2F1 (+) _RAS and the expression of NR2F1 in venous EC1 and venous EC2. **(D)** Correlation analysis between the top 10 transcription factors unique to high MRG score venous EC1 and MRGs. The top 10 transcription factors in high MRG score venous EC show a positive correlation with mitochondrial autophagy genes. **(E)** Correlation analysis between the top 10 transcription factors unique to low MRG score venous EC2 and MRGs. The top 10 transcription factors in low MRG score venous EC show a negative correlation with MRGs.

Utilizing pySCENIC, we explored the transcription factors disparities between two type venous EC exhibiting divergent MRGs scores. Transcription factors notably affiliated with venous EC1 include BCL3(+), RELA (+), NELFE (+), while those intimately associated with venous EC2 encompass NR2F1 (+), BHLHE41 (+), FOXC2 (+) (Figure 6A) (supplement Excel1). We then further investigated the expression patterns of the transcription factors BCL3 and NR2F1 within the two types of venous EC. The score of BCL3(+) _RAS (Regulon Activity Score) and the expression of BCL3 were both ascend in venous EC1; while the opposite situation was observed for NR2F1 (+) and NR2F1 (Figures 6B, C). Moreover, we conducted a correlation analysis at the bulk transcriptome level between the top 10 transcription factors unique to each venous EC and mitochondrial autophagy genes. The outcome revealed that the top 10 transcription factors of high MRGs score venous EC are predominantly positively correlated with mitochondrial autophagy genes (Figure 6D), while those of low MRGs score venous EC are primarily negatively correlated (Figure 6E). These findings underscore the critical role of these transcription factors in modulating mitochondrial autophagy within the venous EC.

### 3.6 Significant disparities exist between high and low MRG scores in ECs on the MIF and TGFb pathways

**FIGURE 7:**
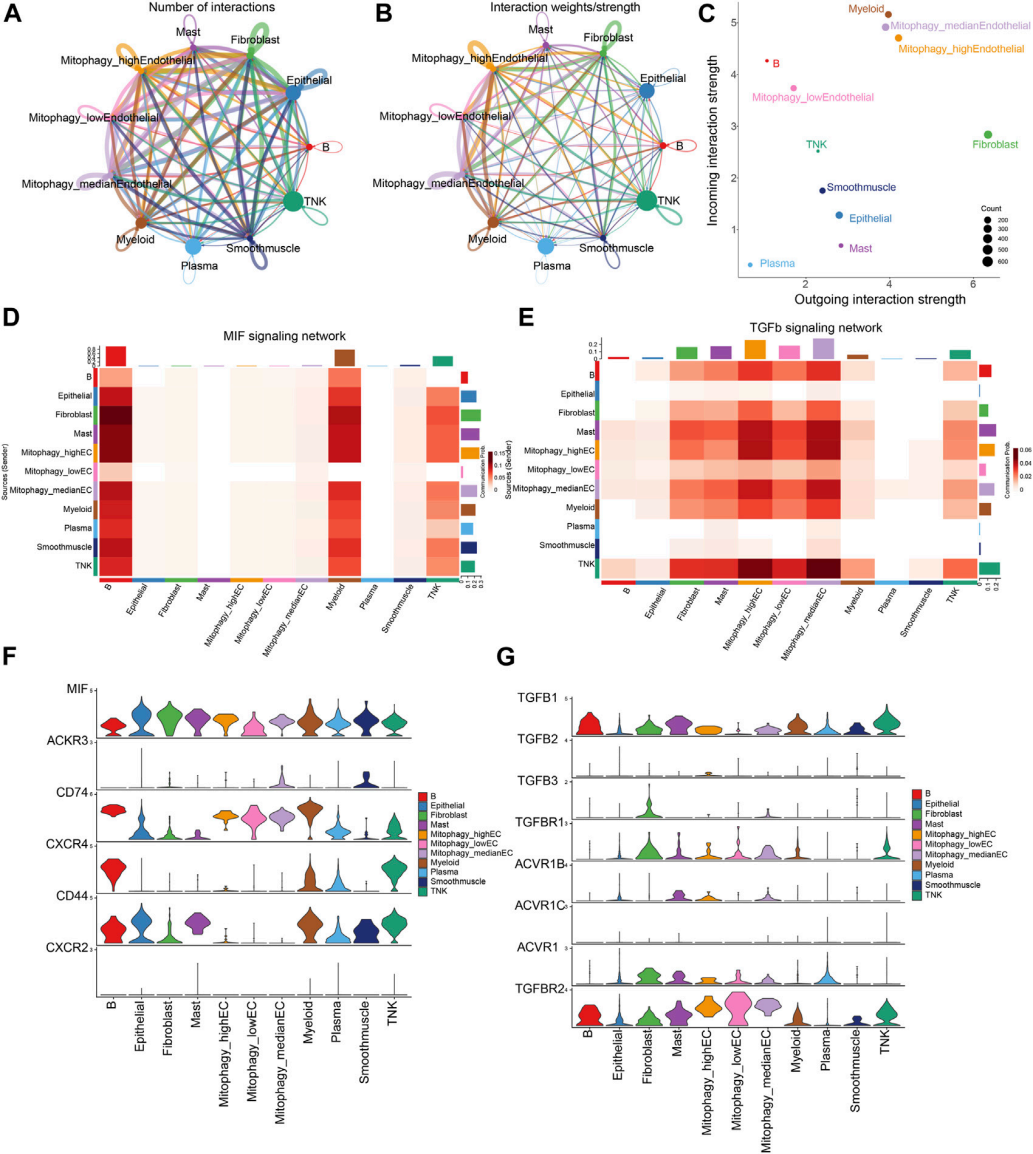
Disparities in intercellular communication and pathway utilization among ECs with varying MRG scores. **(A,B)** Graphic representation of intercellular communication dynamics across various cell types. **(C)** Comparison of signal transduction and reception capabilities between Mitophagy_high ECs and Mitophagy_low ECs. **(D)** Heatmap demonstrating the unique communication capabilities of Mitophagy_high ECs with B cells, myeloid cells, and TNK cells via the MIF pathway, which Mitophagy_low ECs lack. **(E)** Illustration of the diminished communication capacity of Mitophagy_low ECs via the TGFb pathway. **(F,G)** Violin plots representing the expression of MIF and TGFB1 receptors in the MIF and TGFb pathways across various cell types, indicating higher expression in Mitophagy_high ECs compared to Mitophagy_low ECs.

ECs are stratified into three categories based on their MRG scores: Mitophagy_highECs (scoring above the 75th percentile), Mitophagy_lowECs (scoring below the 25th percentile), and Mitophagy_medianECs (with scores falling within the 25th to 75th percentile). We employed CellChat to investigate the differences in intercellular communication among ECs with varying MRG scores and other cells. Figures 7A, B graphically present the intercellular communication dynamics among various cell types. Mitophagy_high ECs demonstrate significant potential for both signal transduction and reception, while Mitophagy_low ECs primarily exhibit high signal reception ability (Figure 7C). We depicted the specific cellular communication scenarios when Mitophagy_high ECs and Mitophagy_low ECs act as signal senders. The results identified a preferential utilization of the MIF signaling pathway in the communication between Mitophagy_high ECs and other cells (Supplementary Figure S4). The heatmap further illustrates that Mitophagy_high ECs can communicate with B cells, myeloid cells, and TNK cells via the MIF pathway, while Mitophagy_low ECs are unable to do so (Figure 7D). We also observed a diminished communication capacity of Mitophagy_low ECs through the TGFb pathway (Figure 7E). Further, we employed violin plots to represent the expression of receptors in the MIF and TGFb pathways across Background Methods Results Conclusionvarious cell types. MIF and TGFB1 genes manifest elevated expression levels in Mitophagy_high ECs compared to Mitophagy_low ECs (Figures 7F, G).

## 4 Discussion

CRS is a common inflammatory condition affecting the paranasal sinuses and nasal mucosa, with a complex pathophysiology involving microbial, immunological, and inflammatory responses (Yao et al., 2023). Despite extensive research, the exact etiology of CRS remains unclear, presenting significant challenges in the diagnosis and management of the condition (Kato et al., 2022). Mitophagy, the selective degradation of mitochondria by autophagy, plays a crucial role in cellular homeostasis, maintaining the quality of the mitochondrial network by eliminating damaged or unnecessary mitochondria (Perrone et al., 2023).

Mitophagy plays a pivotal role in respiratory diseases. In Chronic Obstructive Pulmonary Disease (COPD), the augmented expression of mitophagy-associated proteins can deter the buildup of Reactive Oxygen Species (ROS) and cellular senescence, thereby mitigating the severity of the disease (Araya et al., 2019). Yet, another investigation postulates that enhanced mitophagy could exacerbate COPD (Zhang et al., 2019). In Acute Respiratory Distress Syndrome patients, mitophagy could potentially thwart mitochondria-induced apoptosis, thereby alleviating respiratory symptoms (Li et al., 2019).

For Asthma, contemporary research indicates that the activation of mitophagy could impact the symptoms in asthmatic patients. The expression of BNIP3 is found to be elevated in the airway smooth muscle cells of asthmatic patients (Pan et al., 2019). In parallel, another study suggests that by curbing mitophagy in human bronchial epithelial cells, allergic airway inflammation can be mitigated in asthmatic individuals (Zhang et al., 2021). Mitophagy-associated proteins such as PINK1 and Parkin are overexpressed in fibroblasts procured from asthmatic patients (Ramakrishnan et al., 2020).

Conversely, the role of mitophagy in nasal inflammation is not well defined. Phosphatase and tensin homolog could potentially deter nasal inflammation by restraining mitophagy in nasal epithelial cells (Ramakrishnan et al., 2020). Bleomycin-A5 can suppress dynamin-related protein 1-mediated mitophagy in fibroblasts, leading to apoptosis in nasal polyps (Wu et al., 2021). A recent study revealed that the downregulation of autophagy and mitophagy was noted in eosinophilic and noneosinophilic nasal polyps, exhibiting a negative correlation with the severity of eosinophilic inflammation (Wang C. et al., 2022). In the present era, the swift advancement of scRNA-seq technology offers an innovative approach to investigate CRS (Wang W. et al., 2022). In the present study, we have conducted a comprehensive analysis of MRGs in CRS, using both bulk and single-cell RNA sequencing data to provide an in-depth understanding of the heterogeneity of endothelial cells in CRS.

Through our examination of bulk RNA sequencing data, we unveiled a potential crucial role of MRGs in CRS. Initially, we discerned seven pivotal genes—SQSTM1, SRC, UBA52, MFN2, UBC, RPS27A, and ATG12—displaying differential expression in CRS patients as opposed to controls, and based on these key genes, we established a proficient model for CRS diagnosis. Among the hub MRGs, SQSTM1, a versatile protein, is integral to cellular processes like autophagy and mitophagy, which involve selective mitochondrial degradation (Liu W. et al., 2022).

Notably, SQSTM1 facilitates mitochondrial ubiquitination independent of PINK1 and PRKN/parkin in mitophagy (Yamada et al., 2019). Furthermore, our findings substantiate the association between the expression of pivotal MRGs and the immune microenvironment as well as inflammatory factors. Specifically, a robust positive correlation was observed between the seven central MRGs and resting dendritic cells, contrasted with a notable negative correlation with mast cells and CD8^+^ T cells, thereby underlining the intricate relationship between mitophagy and the immune response in CRS. Simultaneously, we discovered a potent correlation between TGFB1 and MRGs, indicating the significant influence TGFB1 could have on mitophagy in nasal mucosa. TGF-β1 can stimulate mitophagy, thereby promoting the *ex vivo* generation of erythrocytes from hematopoietic stem cells (Kuhikar et al., 2020). Furthermore, TGF-β1 incites mitochondrial ROS and depolarization in lung epithelial cells, as well as stabilizes the vital mitophagy initiating protein, PINK1 (Patel et al., 2015). Lastly, our analysis proposes that CRS can be bifurcated into two subtypes grounded on MRG expression patterns, unveiling substantial heterogeneity in MRG expression across the two clusters. These revelations could potentially carry weighty implications for patient categorization and tailored treatment strategies in CRS.

Our comprehensive analysis of MRGs and EC heterogeneity in Chronic Rhinosinusitis (CRS) in single cell levels enhances the understanding of this complex inflammatory condition. The expression of the hub genes in different cell types. Among them, UBC has the highest expression level in EC. Some research delves into the role of mitophagy in EC amidst oxidative stress and energy scarcity (Li C. et al., 2022; Coon et al., 2022; Wen et al., 2023). ECs with damaged mitochondria are removed by mitophagy, which lessens cellular damage (Rong et al., 2021; Li A. et al., 2022). Furthermore, when ECs encounter hemin, a lipid peroxidation cascade ensues, triggering mitochondrial depolarization and subsequent mitophagy (Basit et al., 2017).

Furthermore, we identified two venous EC types with distinct MRG scores, suggesting their different role in the pathogenesis of CRS, particularly the neCRSwNP subtype. Venous EC1 exhibited high MRG scores and a higher proportion in neCRSwNP, alongside activation of the mitophagy pathway. These cells were enriched in “Epithelial-Mesenchymal Transition”, “Notch Signaling”, and “Protein Secretion” pathways, suggesting their involvement in tissue remodeling, immune regulation, and mucus production. Venous EC2, conversely, had lower MRG scores, were underrepresented in neCRSwNP, and exhibited suppressed mitophagy. They were enriched in “Wnt Beta-Catenin Signaling”, “PI3K Akt mTOR Signaling”, and “IL6 Jak STAT3 Signaling” pathways, indicative of their role in inflammation and tissue repair. Further, we unveil that unique transcription factor profiles were discerned for both categories of venous endothelial cells (ECs). A noteworthy positive correlation was observed between the paramount transcription factors (BCL3(+)) and mitophagy genes in Venous EC1 s, insinuating their significant role in regulating mitochondrial autophagy.

Intercellular communication analysis demonstrated significant disparities between high and low MRG scoring ECs. Mitophagy_high ECs primarily utilized the MIF signaling pathway, while Mitophagy_low ECs demonstrated diminished communication via the TGFb pathway. The TGFb pathway plays a multifaceted role in CRS pathogenesis, influencing tissue remodeling, inflammation, immune regulation, and mucociliary clearance. Dysregulation in this pathway can exacerbate CRS symptoms, making it a potential therapeutic target (Lai et al., 2022). The MIF pathway significantly influences chronic rhinosinusitis (CRS) by modulating inflammation, tissue remodeling, and mucosal barrier function. Elevated MIF levels in CRS patients suggest its role in persistent sinus inflammation (Yuan et al., 2021). These findings suggest the role of MIF and TGFb pathways in mediating mitophagy’s effects in CRS. Although the precise mechanisms remain nebulous, the MIF signaling trajectory indeed plays a pivotal role within the context of mitophagy (El Bounkari and Bernhagen, 2012). Established investigations have discerned and explicated two distinct signaling trajectories of mitophagy: those contingent upon ubiquitin (Ub) and those independent thereof, the latter of which are facilitated via receptor-mediated mechanisms (Harris et al., 2018). Certain scholarly discourse posits that Migration Inhibitory Factor (MIF) instigates mitophagy via the BNIP3-mediated conduit, a modality of mitophagy that operates independently of ubiquitin (Lai et al., 2015). Conversely, alternate perspectives argue that MIF presides over the regulation of PINK1/Parkin-mediated mitophagy, representing the most comprehensively characterized ubiquitin-dependent pathway of mitophagy (Smith et al., 2019). Specifically, deficiency of MIF Accentuates Overloaded Compression-Induced Nucleus Pulposus Cell Oxidative Damage via Depressing Mitophagy (Wang et al., 2021).

This study stands out as one of the pioneering investigations into the role of endothelial dysfunction and mitophagy in CRS. By utilizing both bulk and single-cell RNA sequencing data, we have obtained a granular insight into the molecular dynamics at play. This approach allowed us to unravel previously overlooked aspects of CRS and provided a holistic view of the endothelial changes in the condition. However, we acknowledge several limitations in our research. Primarily, the results might not comprehensively represent the endothelial functional status across all CRS patients due to sample sourcing and technological constraints. Secondly, while our study emphasizes the role of mitophagy in CRS endothelial dysfunction, mechanistic studies are required to further confirm these findings. Lastly, our conclusions are primarily based on RNA sequencing data. Future studies employing proteomics and functional assays would be invaluable to verify and extend our findings. In conclusion, our comprehensive investigation of CRS elucidates the significant role of MRGs and highlights the heterogeneity of ECs. We identified key MRGs, revealing their interplay with immune responses and inflammation, and underscored the heterogeneity within CRS subtypes, informing patient stratification and personalized treatment. At the single-cell level, venous ECs displayed distinct MRG scores, hinting at their differential roles in CRS pathogenesis. We spotlighted the importance of MIF and TGFb pathways in mediating mitophagy’s effects, especially the MIF. Overall, our findings enhance the understanding of mitophagy in CRS, paving the way for future research and therapeutic advancements.

## Data Availability

The datasets presented in this study can be found in online repositories. The names of the repository/repositories and accession number(s) can be found in the article/Supplementary Material.

## Supplementary material

SUPPLEMENTARY FIGURE S1
**(A)** The PCA graph before batch removing. **(B)** OR value and confidence interval of key genes. **(C)** Distribution range of under area of AUC. **(D)** Distribution range of sensitivity. **(E)** Distribution range of specificity.

SUPPLEMENTARY FIGURE S2
**(A)** Clustering dendrograms of samples. **(B)** Analysis of network topologies for various soft-thresholding powers through the scale-free fit index (left) and mean connectivity (right).

SUPPLEMENTARY FIGURE S3 The Ucell scores among groups for all cell types.

SUPPLEMENTARY FIGURE S4 the specific cellular communication scenarios when Mitophagy_high ECs and Mitophagy_low ECs act as signal senders.
