# Supplementary material for: Comprehensive analysis of mitophagy-related genes in diagnosis and heterogeneous endothelial cells in chronic rhinosinusitis: based on bulk and single-cell RNA sequencing data
Source: Front Genet. 2023 Sep 8;14:1228028. doi: 10.3389/fgene.2023.1228028 (PMC10514917; doi:10.3389/fgene.2023.1228028)
Supplement: Supplementary file 2 [file Presentation1.pdf]

Supplementary Material

Supplementary Figures and Tables

Supplementary Figures

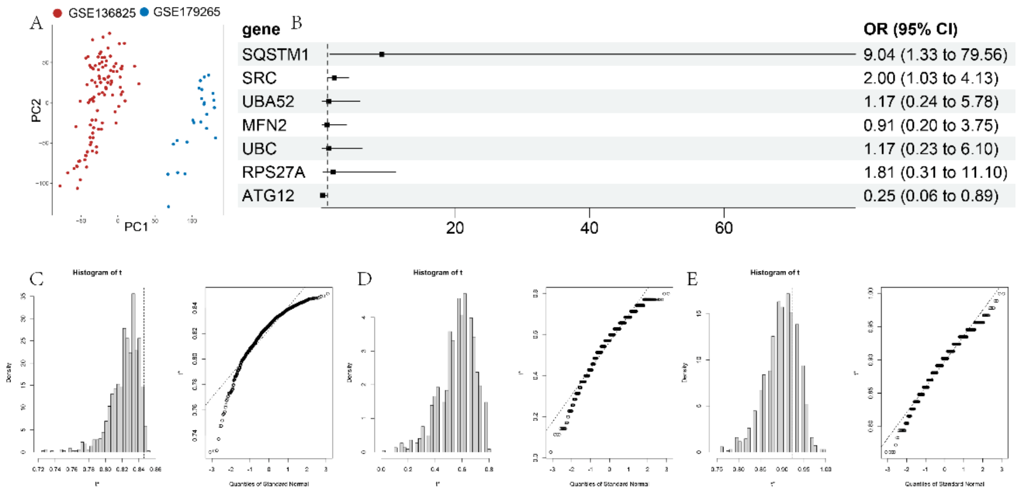

Supplementary Figure 1 (A) The PCA graph before batch removing. (B) OR value and confidence interval of key genes. (C)Distribution range of under area of AUC. (D) Distribution range of sensitivity. (E)Distribution range of specificity.

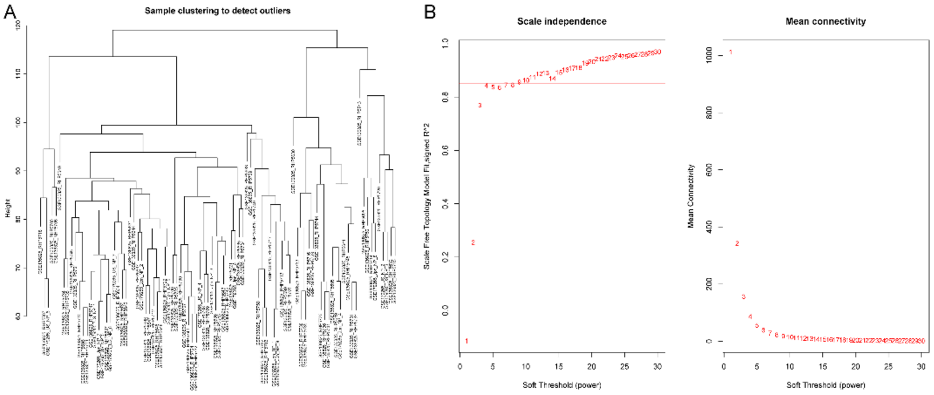

Supplementary Figure 2 (A) Clustering dendrograms of samples. (B) Analysis of network topologies for various soft-thresholding powers through the scale-free fit index (left) and mean connectivity (right).

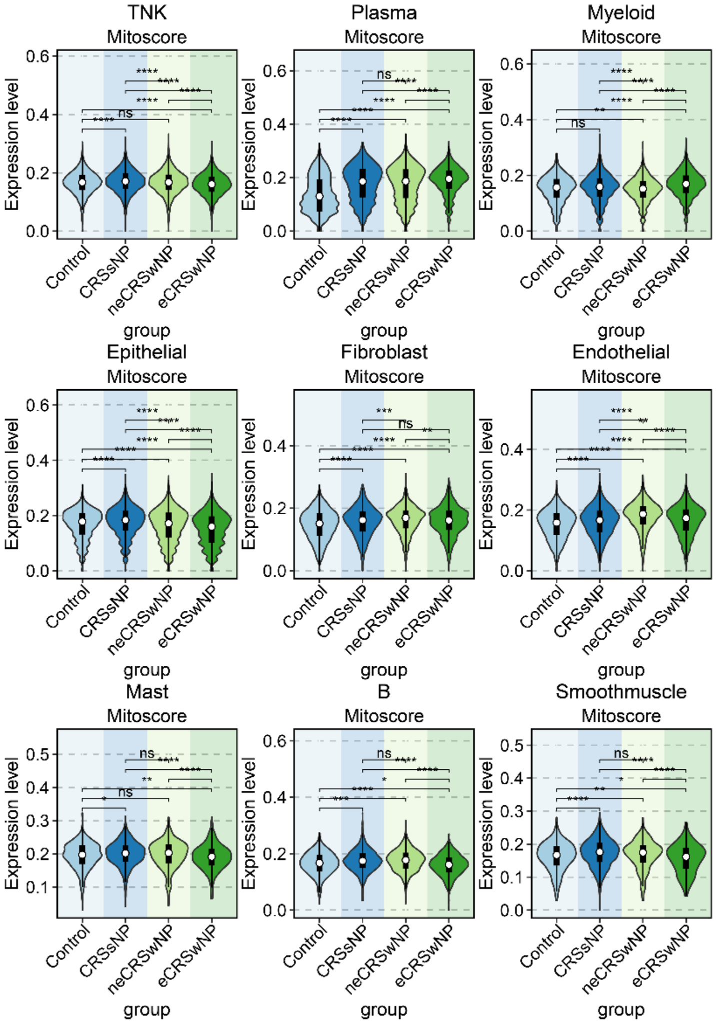

Supplementary Figure 3 The Ucell scores among groups for all cell types.

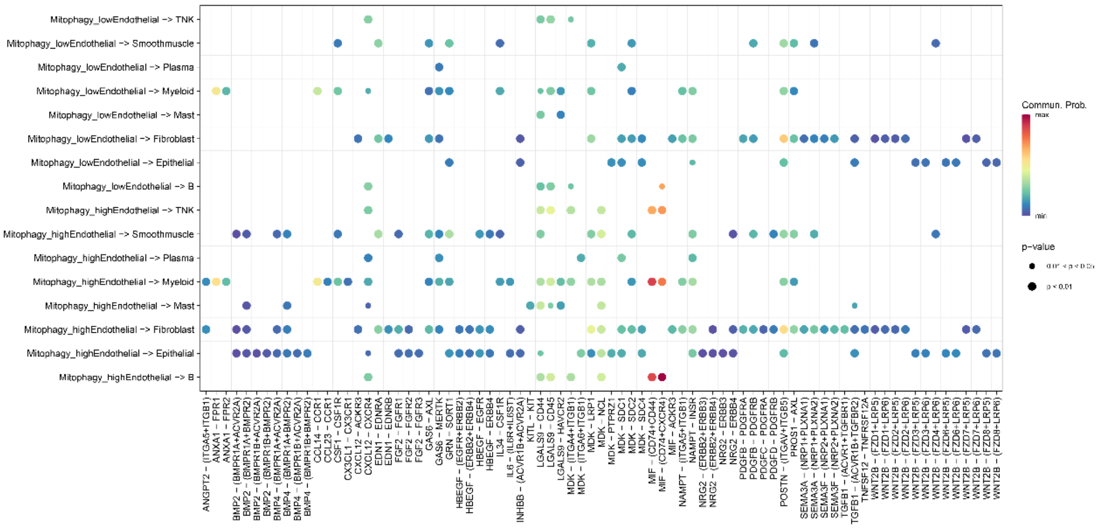

Supplementary Figure 4 the specific cellular communication scenarios when Mitophagy_high ECs and Mitophagy_low ECs act as signal senders.
