# Supplementary figures and images for: Comprehensive analysis of mitophagy-related genes in diagnosis and heterogeneous endothelial cells in chronic rhinosinusitis: based on bulk and single-cell RNA sequencing data
Source: Front Genet. 2023 Sep 8;14:1228028. doi: 10.3389/fgene.2023.1228028 (PMC10514917; doi:10.3389/fgene.2023.1228028)

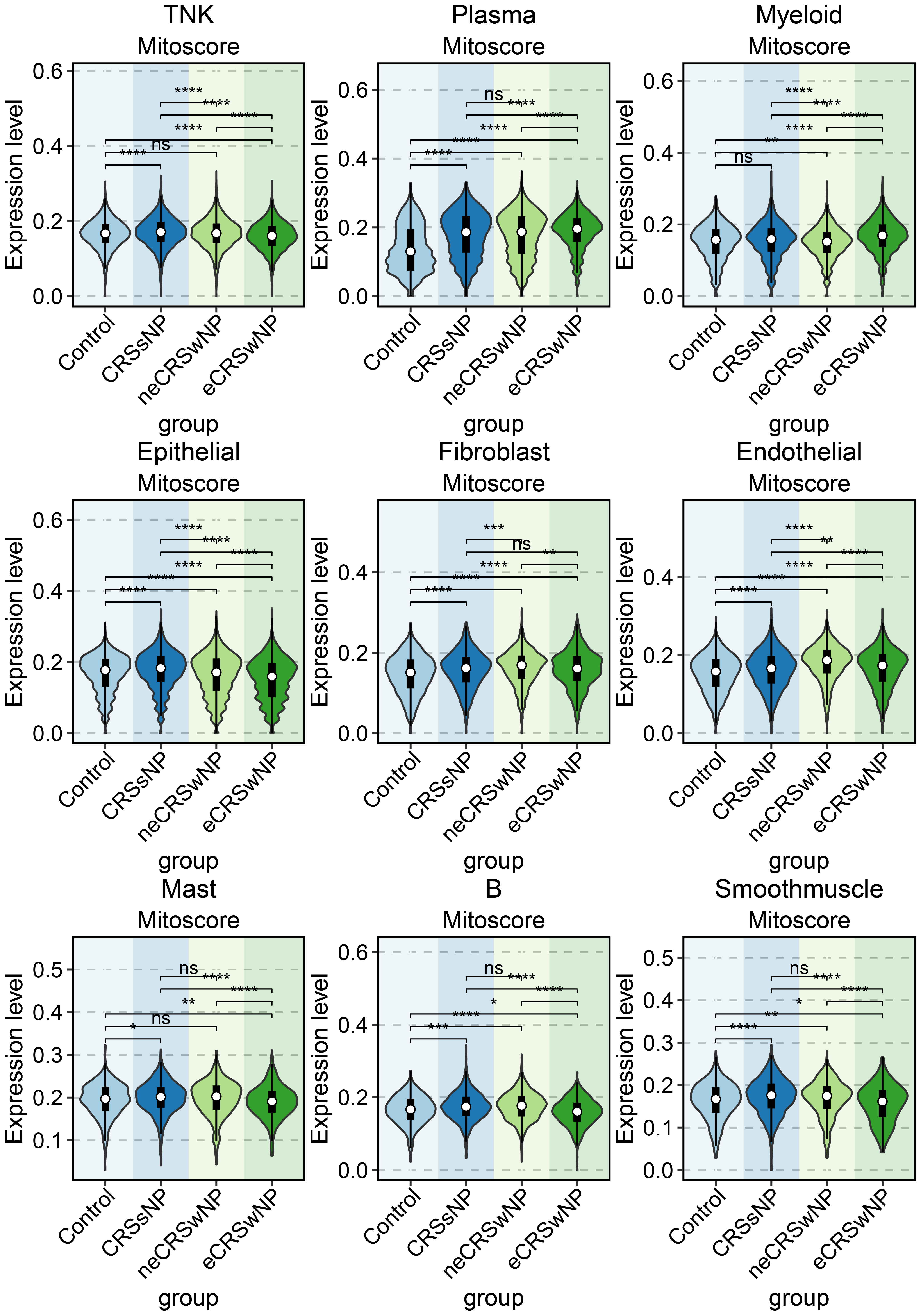

Supplement: Supplementary file 3 [file Image3.TIF]

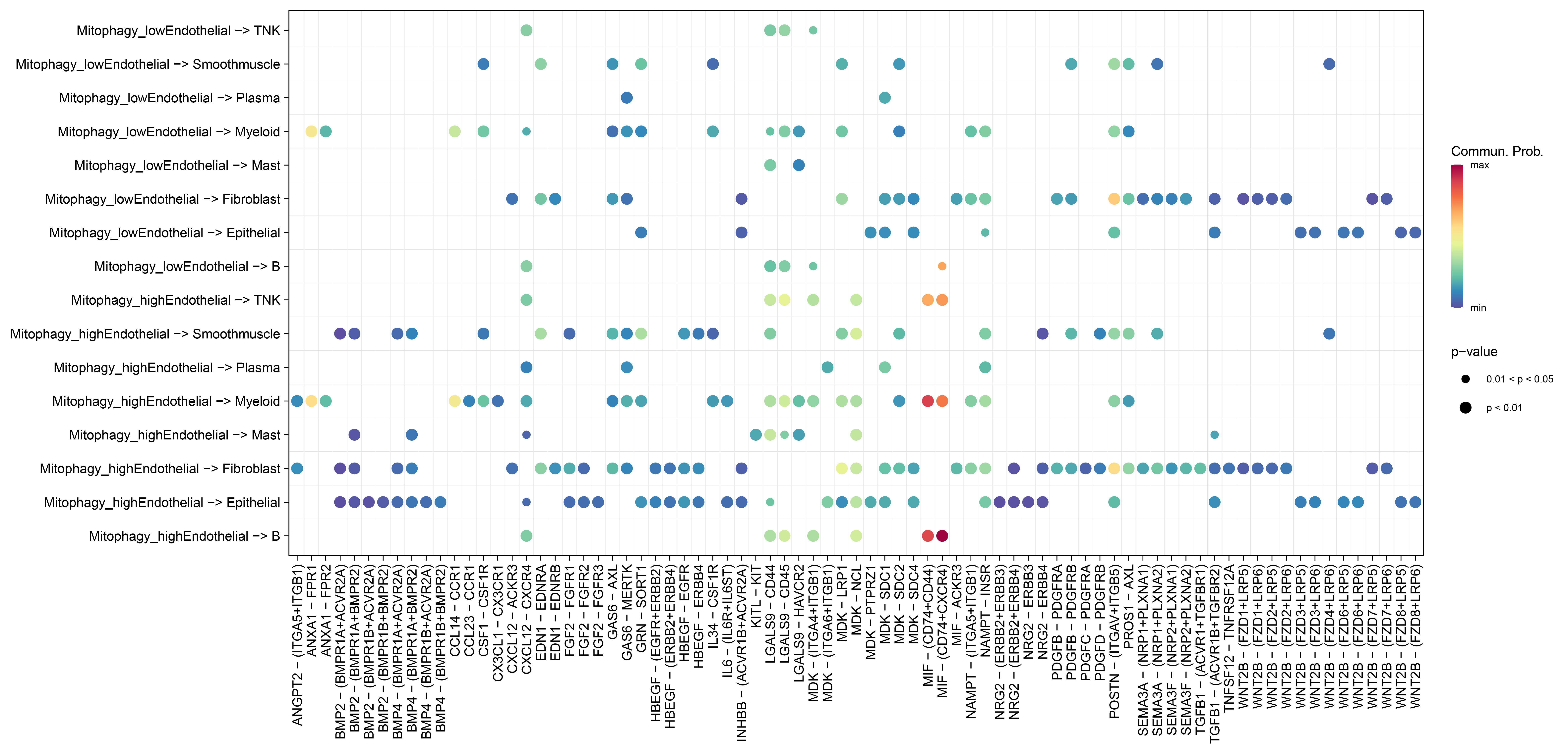

Supplement: Supplementary file 4 [file Image4.TIF]

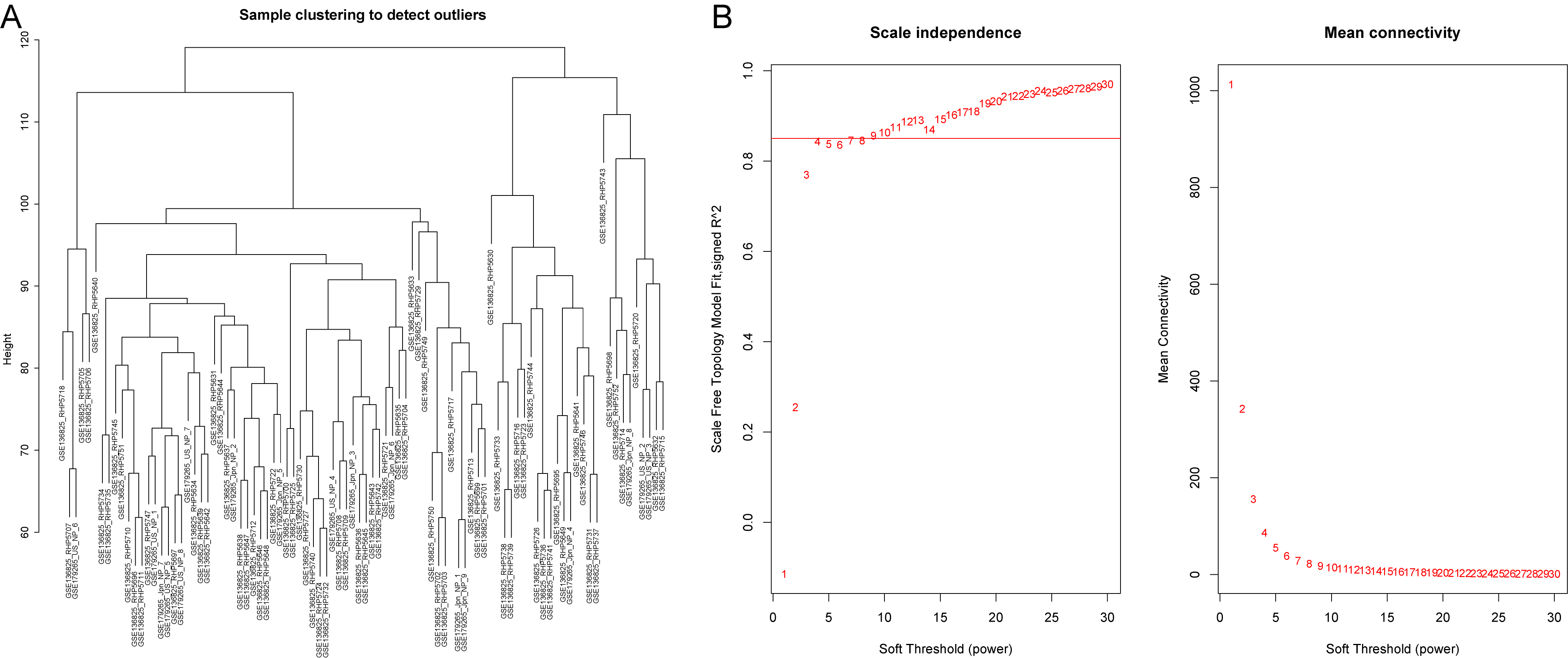

Supplement: Supplementary file 5 [file Image2.TIF]

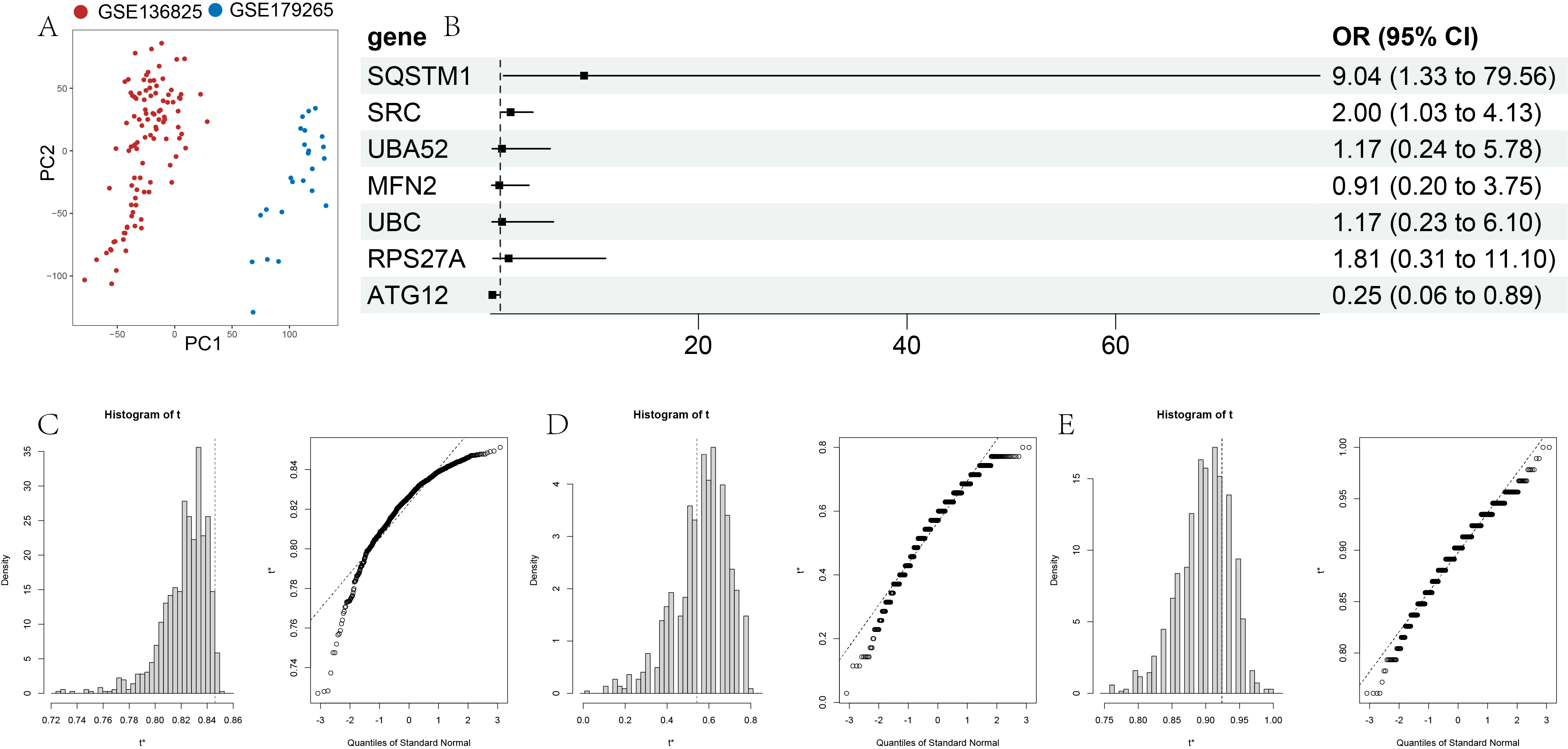

Supplement: Supplementary file 6 [file Image1.TIF]
